# Application of Top-Down and Bottom-up Systems Approaches in Ruminant Physiology and Metabolism

**DOI:** 10.2174/138920212801619269

**Published:** 2012-08

**Authors:** Khuram Shahzad, Juan J Loor

**Affiliations:** 1 Department of Animal Sciences, University of Illinois, Urbana-Champaign, Urbana, Illinois, 61801, USA

**Keywords:** Bioinformatics, dairy cow, nutrigenomics, microarray.

## Abstract

Systems biology is a computational field that has been used for several years across different scientific areas of biological research to uncover the complex interactions occurring in living organisms. Applications of systems concepts at the mammalian genome level are quite challenging, and new complimentary computational/experimental techniques are being introduced. Most recent work applying modern systems biology techniques has been conducted on bacteria, yeast, mouse, and human genomes. However, these concepts and tools are equally applicable to other species including ruminants (e.g., livestock). In systems biology, both bottom-up and top-down approaches are central to assemble information from all levels of biological pathways that must coordinate physiological processes. A bottom-up approach encompasses draft reconstruction, manual curation, network reconstruction through mathematical methods, and validation of these models through literature analysis (i.e., bibliomics). Whereas top-down approach encompasses metabolic network reconstructions using ‘omics’ data (e.g., transcriptomics, proteomics) generated through DNA microarrays, RNA-Seq or other modern high-throughput genomic techniques using appropriate statistical and bioinformatics methodologies. In this review we focus on top-down approach as a means to improve our knowledge of underlying metabolic processes in ruminants in the context of nutrition. We also explore the usefulness of tissue specific reconstructions (e.g., liver and adipose tissue) in cattle as a means to enhance productive efficiency.

## INTRODUCTION

Systems biology is an interdisciplinary field that concentrates on experimental and computational biology. At the core of this approach, which is not novel, is the concept of dealing with a system as a whole rather than its constitutive parts. Advancements in computational biology, genome sequencing, and high-throughput technologies in the last decade have increased the awareness of the scientific community for approaching biological systems in an integrative fashion, i.e. allow access to the functional capabilities of an individual organism en masse. However, the notion of dealing with a system as a whole was proposed several decades earlier. For instance, in 1934 the Austrian biologist Ludwig von Bertalanffy proposed the application of the “general systems theory” (GST) in biology, cybernetics (structural study of regulatory systems) and other areas [1]. In the mid-20th century, the geneticist and biochemist Henrik Kacser focused on the use of systematic approaches instead of analyzing separate components of a metabolic system [2]. Mihajlo Mesarovic (1968), a mathematician and engineer at Case Western Reserve University, also emphasized the need for systematic applications in biology [3].

The field of genomics and transcriptomics has already provided an enormous amount of biological information. Currently, there is a need to communicate biological knowledge systematically, e.g., linking the genome to the whole organism. Newly emerging bioinformatics techniques along with biological data generated from genomics and transcriptomics studies have already allowed biologists to apply modern systems approaches to study interactions occurring inside living systems. The work of Palsson’s group from the 1990’s onward contributed to the development of genome-scale mathematical models to understand the biological interactions from simpler organisms (e.g., microbes) to humans. From 1999 onward, with the first genome-wide metabolic reconstruction of *Haemophilus influenza* [4], research in the field of modern systems biology has exploded. Several genome-wide and tissue-specific reconstruction projects across a broad range of species have been published, e.g., more than 50 in 2009 [5] to more than 80 in 2011. It is likely that work in this area will continue to grow. Currently available genome-scale metabolic reconstructions ranging from bacteria, archaea, to multicellular eukaryotes are shown in Fig. (**1**). (Retrieved from Systems Biology Research Group, University of California San Diego; on June 19, 2011 [http://systemsbiology.ucsd.edu/In_Silico_Organisms/Other_Organisms]).

Genome-scale metabolic network reconstructions of model organisms have been assembled in a BiGG (biochemically, genetically, and genomically structured) knowledgebase [6] that aims to represent all known metabolic pathways of an organism. The BiGG knowledgebase works with the COBRA (constraint based reconstruction and analysis) toolbox [7], while metabolic network reconstructions hosted by it are created using the steps described in details by Reed *et al.* [8], Feist *et al.* [9] and Thiele and Palsson [10]. These reconstructions have been assembled for more than 80 different organisms ranging from unicellular (e.g., bacteria [4, 11] and yeast [12]), to multicellular organisms (e.g., mouse [13], Arabidopsis thaliana [14], and humans [15, 16]).

The expanding suite of tools for applying modern systems biology requires bioinformatics expertise. Bioinformatics is generally defined as a field that relies on computational resources to analyze biological data (e.g., genome, transcriptome, metablome, or fluxome) on a large scale [17]. It also encompasses the development of tools ranging from genome to proteome analyses including transcriptomics data [18, 19]. One of the goals of bioinformatics is to accelerate the interpretation of large amounts of ‘omics’ data [19]. For instance, Lemay *et al.* [20] applied this technique on mouse mammary tissue microarray data that was generated during pregnancy, involution and lactation time points.

With the rapid development of bioinformatics analysis tools, there is a need to tailor some of those to help in the automation of ruminant genomics. From a ruminant animal perspective, one long-term goal of this process involves the development of mathematical and mechanistic models that would link the genome (e.g., bovine, caprine) to the whole organism [21]. The pioneering work of Baldwin and his colleagues [22-24] provided one of the first comprehensive mathematical models (‘Molly’) that attempted to link genotypic to phenotypic data [25]. The model was aimed at determining the relationship between diet and animal performance [26]. In essence, the goal was to develop “simple” models to understand the relationship between digestive processes and their effects on metabolic pathways in liver, mammary, and adipose tissue of dairy cattle (*Bos taurus*). Upon successful completion of the cattle genome sequencing project [27], the process of genome-wide and tissue-specific reconstructions in this species was accelerated with the application of both “top-down” and “bottom-up” approaches. An initial attempt to assemble genome-wide metabolic pathway information has already been performed by Seo and Lewin [28]. Further information about these metabolic pathways can be found using the online BioCyc and MetaCyc databases [29-31].

The aim of this review is to provide a brief description of modern systems biology concepts and their applications in high-producing ruminants (i.e., dairy cattle). We succinctly describe the top-down and bottom-up approaches but mainly focus on the top-down approach for metabolic pathways reconstruction and analysis. The overall goal is to underscore the uniqueness of these approaches to provide a holistic view of complex biological interactions occurring in ruminants. We also discuss current methodologies that would help to accelerate metabolic reconstruction in ruminants as a means to enhance our biological and practical knowledge. In particular, we provide tissue-specific examples of ongoing efforts in the top-down reconstructions in the bovine. We believe that such knowledge will, in the long-term, help to improve efficiency of nutrient use in particular, and contribute in meeting the growing needs of high-quality food for human consumption.

## MODERN SYSTEMS BIOLOGY

Modern systems biology refers to the use of both mathematical and ‘omics’ approaches to expand the knowledge of biological functions [32]. In this context, one of the widely-accepted approaches for mathematical modeling is the use of constraint-based modeling established by Price *et al.* [33]. Within this approach, constraints are applied under mathematical frameworks to mimic real-life biological activities (e.g., the interaction between reactants and products) *in silico*. These constraints implicitly define the solution space of a metabolite and its reactions with respect to other metabolites. The solution space is a mathematical term that can be defined using biological phenomena such as an allowed region in a biological network where reactants can be converted into one or more possible products [33]. During such conversions a steady-state flux distribution is required through all the reactions. These steady-state flux distributions are described in terms of extreme pathways whereas these extreme pathways are categorized into three main types that measure the flux distributions among the participating substrates, cofactors, and products during a series of reaction steps [34, 35].

The detailed methodology of constraint-based modeling was developed into a computational tool called COBRA by Becker and his colleagues [36]. The COBRA toolbox is widely used in systems biology to reconstruct genome-scale mathematical models. This toolbox performs flux-balance analysis (FBA) that is used to define the metabolic behavior of substrates and their products within a solution space context [37, 38]. Recently, this tool is further modified into a new version 2.0 by Schellenberger *et al.* [7] to contain improved functions such as “network gap filling, ^13^C analysis, metabolic engineering, omics-guided analysis, and visualization”. This tool has facilitated efforts to integrate biological systems, effectively expanding from the reductionist methodologies.

The reconstructed mathematical models are used to simulate user-defined biological conditions *in silico*. For example, these models can be used in drug designing [39], biofuel production [40], or in numerous other related applications. An important focus of systems biology has been to uncover new characteristics emanating from the network interactions, all of which should lead to a more holistic view of an organism [19] and its useful applications for the benefits of humans. This emerging field also is dedicated to understanding the physiology of normal and abnormal (diseased) states from a cellular level to the whole organism [18].

## SYSTEMS BIOLOGY APPROACHES

The metabolic behavior of a cell can be approached in either a bottom-up or top-down directionality. The former encompasses the development of automated tools and implementation of mathematical models; whereas, the latter encompasses data processing from ‘omics’ levels to pathways and/or individual gene levels of an organism [41]. Oltvai and Barabasi depicted these approaches in the form of a pyramid describing two different levels in terms of “organism specificity” and “universality”. They emphasized that a cell can be approached from both bottom to top (universality) or from top to bottom (organism specificity) equally, i.e., from molecules to the scale-free networks or modules, or moving from a network scale-free and hierarchical nature to organism-specific modules [42]. In contrast, Kummel *et al.* [43] combined these two sets of approaches with the second law of thermodynamics under the name of “network embedded thermodynamics (NET) analysis”. NET analysis essentially combines these three ideas into a single approach to reveal functional behavior of the metabolic network interactions. This is indeed a novel approach to deal with biochemical properties in terms of physical laws of thermodynamics and aimed to help us improve our knowledge of cell physiology. There also are ongoing efforts for building automated tools that incorporate the steps of the bottom up approach to automatically create genome-scale models. One example is the availability of a software called SEED which was initially validated with *Staphylococcus aureus* [44].

### A) Bottom-up Approach

The bottom-up approach is aimed at thoroughly crafting detailed models that can be simulated under different physiological conditions. This approach combines all organism-specific information into a complete genome-scale model to provide an integrative view of the biological interactions occurring inside living systems. It employs the methodology built on constraint-based modeling [33], that allows to build genome-scale mathematical models using four main steps, which are i) draft reconstruction, ii) manual curation, iii) converting curated models into mathematical format, and then iv) validation of these models using literature reviews (bibliomics data), biochemical assays, and ‘omics’ data [9, 10]. These four steps are summarized below:

#### i) Draft Reconstruction

Draft reconstruction encompasses data collection from different online resources such as genomics, biochemical, metabolic, and/or organism-specific databases. The data are extracted through bioinformatics software tools e.g., pathway tools [45] and metaSHARK [46]. In the case of ruminant draft reconstruction projects, freely accessible genomics databases include NCBI [47], EntrezGene [48], UCSC Genome Browser [49], UniPort [50] and BGD (Bovine Genome Database) [51]; biochemical databases include KEGG (Kyoto Encyclopedia of Genes and Genomes) [52], BRENDA (BRaunschweig ENzyme DAtabase) [53, 54], PubChem identifier [55], CAS (Chemical Abstracts Service) [56], CheBI (Chemical Entities of Biological Interest) [57], and Transport DB [58]; and among the metabolic- and organism-specific reconstruction databases are (but not limited to) Reactome [59], BioCyc and MetaCYC [29-31]. Draft reconstruction is an automated process; hence, there are equally likely chances of incorporating incorrect information of metabolites or failing to include key metabolites or their reaction information [10]. To avoid this misrepresentation, further manual curation is required, which is briefly described in the following step.

#### ii) Manual Curation

This step is human-intensive and dependent on the actual organism-specific genome, metabolome, or fluxome information. Software-assisted (e.g., pathway tools) draft construction steps help to add missing data or to remove unnecessary information. To validate the constructed draft, textbooks, scientific articles, literature reviews, biochemical assays (i.e., validation), and organism-specific databases are used [9, 10]. For ruminant-specific reconstructions, knowledge of metabolic pathway conservation relative to other mammals (e.g., mouse, human) is also useful. For example, evolutionary divergence of metabolic pathways can be helpful to uncover similarities and differences between the organism of interest (e.g., bovine) and known organisms (e.g., human) to build a common evolutionary relationship. This illustration can be exemplified using the creation of fish metabolic network (MetaFishNet) [60]. This metabolic network is built upon homology-based searches using relationships from diverse species.

#### iii) Conversion to Mathematical Models

Following the completion of a curated draft, it is transformed into a mathematical language to perform simulations. For this purpose, mathematical software tools such as Matlab (Mathwork, Natwick, MA, USA) embedded COBRA toolbox [36], SBML (systems biology markup language) software [61], and linear programming (LP) or quadratic programing (QP) solver can be used. During this step, balanced stoichiometric matrices are constructed, biomass objective functions [62] are defined, FBA [38] is performed, and then flux variability analysis (FVA) is conducted to verify the robustness of the model [63].

#### iv) Network Validation

The fourth and final step involves the iterative refinement of the model using different gap-filling algorithms. The model is checked for inconsistencies using defined objective functions. If a reconstructed model is not consistent with the expected results, then the draft is rechecked from step 2 and necessary changes are made. Due to the missing metabolic knowledge in some species, such as gaps (a missing reaction that consumes or produces a metabolite) and orphan reactions (reactions with incomplete or absent information about genes or enzymes), this approach faces some real challenges. [10]. These gaps and orphan reactions can be treated by implementing several gap-filling algorithms described by Orth and Palsson [64]. However, in version 2.0 of COBRA toolbox, gap-filling properties are also included. Following these metabolic network reconstructions, condition-specific models can be derived from a single reconstruction [65]. Fig. (**2**) represents the summary of these four steps.

### B) Top-down Approach

The top-down approach originates from experimental data and information is spanned to reconstruct metabolic models. It can help to unravel biological behavior and underlying interactions using ‘omics’ data, which can be obtained via standard top-down methodologies such as DNA microarrays [66], RNA-Seq [67], or other genome-enabled technologies. According to Van Dien and Schilling [32], the flow of information in the top-down approach occurs from the transcriptome and proteome to flux-balanced metabolic pathways. This approach covers the whole genome; thus, it is considered as a “potentially complete” approach in that it deals with all the genome-wide transcriptomic information [41, 68]. From our perspective, the top-down approach can be explicitly divided into the following five stages. We have presented these stages using the existing DNA microarray case studies Fig. (**3**):

### Stage 1: Sample Collection and Laboratory Experiments

Experiments are designed such that animals are allowed sufficient amounts of time for specific treatments or stimuli to have their effects on selected physiological parameters (e.g., milk production, growth, or fat deposition). More comprehensive studies involve repeated sampling of the same animal over extensive periods of time (e.g. the lactation cycle in dairy cattle or the neonatal period in calves). At the end of a suitable treatment period, tissue samples are collected (e.g., via biopsy or at slaughter) from control and treated animals. Some experiments may not necessarily deal with a treatment per se, but may involve evaluation of ontogenic changes of the transcriptome, proteome, metabolome, or fluxome (e.g. during the lactation cycle). After sample collection, RNA is extracted for subsequent analyses. The RNA extraction protocols may vary, but for most experiments, these involve reagents containing phenol and are based on a classical method developed by Chomczynski and Sacchi [69]. The purification steps involve the use of commercial columns, while extra impurities including residual DNA (if acid phenol-chloroform is not used during extraction) are removed using a commercial DNase I enzyme. The extracted RNA is then reverse-transcribed to cDNA or cRNA and subsequently used for hybridization to DNA, oligonucleotide, or other types of expression microarrays.

### Stage 2: Microarray Platform

DNA microarrays are widely used to determine the expression level of mRNA in specific cell or tissue types. Custom microarray platforms or commercially available platforms, such as Affymetrix [67], Agilent [68], and Amersham BioSciences [69] are generally used. Each microarray slide contains a fixed number of spots, and each spot represents a particular gene. The experiment is performed according to standard protocols mainly involving cDNA synthesis via reverse transcriptase polymerase chain reaction (RT-PCR) from extracted RNA, labeling with fluorescent dyes (e.g., Cy3 and Cy5), hybridization to the arrays, washing, and then scanning of these arrays using confocal laser scanners [70-73]. After scanning array images, data are readily available for normalization and statistical analysis.

### Stage 3: Statistical Analysis

Before employing the standard statistical analysis, data are preprocessed by using one of several available normalization techniques to remove systematic bias while preserving the variation in gene expression occurring due to biologically relevant or treatment-related changes in transcription. Data are usually normalized by log-transformations (e.g., log base 2). Following log-transformations, fold-change values can be calculated relative to a control sample or to some reference time point. Subsequently, statistical tests (e.g., paired student t-test [74]) can be applied using statistical software such as SAS (Statistical Analysis System [75]) or R ( Statistical Computing Language [76]). The statistical probability values (*p*-values) to determine differentially expressed genes (DEG) are obtained and adjusted for multiple comparisons using correction methods such as Bonferroni [77] or Benjamini and Hochberg’s false discovery rate (FDR) [78, 79].

### Stage 4: Implementation of Bioinformatics

Microarray (genes/oligonucleotides) inserts/spots are annotated using different databases such as NCBI [47], DAVID [80], or bioDBnet [81]. Annotation helps discern the DEG affected by a particular stimulus or stimuli (e.g. dietary treatments, drug effects, or biological or developmental time points). Typically the FDR probability value cutoff criterion less than 1% (*p *≤ 0.01) or 5% (*p *≤ 0.05) is used to determine DEG. After selecting the list of DEG, bioinformatics software tools are applied to determine the functional significance of affected genes. There are several software packages for microarray data analyses and interpretation ranging from commercial (e.g., MAS 5.0 from Affymetrix platform; Ingenuity Pathway Analysis®) to open-source software (e.g., R bioconductor). According to a survey conducted by Huang and colleagues in 2009 [82] there are approximately 68 bioinformatics enrichment-analysis tools, which are available for curating DEG lists. Among these tools, the DAVID bioinformatics resource is a popular and user-friendly tool to extract biological information from large gene or protein lists [80]. This resource has multiple applications including annotation of large gene lists, function prediction, and function categorization within “chromosomes”, “KEGG pathways” “biological processes”, “cellular components” and “molecular functions”.

To further analyze the biological interactions or pathways, DEG lists can be mined with software tools as implemented in several research projects such as GeneSpring GX [83] is used by Loor *et al.* [70, 71], Ingenuity Pathway Analysis® [84], used by Loor *et al.* [84], and Genesis [85] used by Graugnard *et al.* [86]. Our research group also has recently developed a novel approach termed the dynamic impact approach (DIA) [87, 88] for functional analysis of expression profiling data. The KEGG database [89] is used to visualize the DEG by uploading the list of gene IDs and their respective fold-change values to the KEGG array tool. Ultimately, the goal of these tools is to provide a visualization of the genes and their interactions [90], protein-protein interaction networks [91], or more recently, the dynamic evaluation of changes in metabolic pathways evaluated in terms of overall impact or flux [92]. (Table **1**) provides a list of most commonly used tools for the systematic study of ruminant expression profiling data.

### Stage 5: Data Interpretation and Knowledge Discovery

Following the bioinformatics analyses, the resulting pathway and network data are evaluated by using available scientific articles and organism-specific databases. Heat maps also can be generated from the expression profiling results obtained through DNA microarrays, RNA-Seq or other high-throughput technologies to provide a compact view of the ‘omics’ data [93]. These heat maps of DEG provide results in the form of gene clusters, which could represent an evolutionary relationship among closely and distantly related genes in the genome [94]. Despite the multitude of tools available, there is still a need to develop bioinformatics resources that provide more biologically relevant meaning to the ruminant data. Our group developed the DIA particularly for dealing with the functional analysis of time-course experiments. The approach takes into account the magnitude and significance of change in DEG [87]. Fig. (**3**) summarizes the above five stages of the proposed top-down systems biology approach in ruminants.

As the top-down approach deals with the whole genome, it is considered as a potentially complete approach [41]. There also are certain limitations [95] in this approach; however, the major advantage of this approach is that it provides a more precise view of the fate of metabolites. Hence, it can help us to understand the molecular behavior (e.g., metabolism, signaling, transport) of genes or proteins under certain environmental or dietary conditions and physiological states, such as parturition (stressed condition), and negative energy balance in the post-partum period [96].

### The Role of Systems Biology in Ruminant Metabolism and Physiology

Within the context of nutrient usage as it relates to physiology, ruminant systems biology focuses on the systematic study of complex biological interactions occurring in different tissues that are directly (mammary) or indirectly (liver, muscle, adipose tissue) involved in coordinating physiological adaptations, and particularly susceptible to nutritional management. Recent advances in bioinformatics and systems biology techniques have accelerated the genome-wide and tissue-specific reconstruction to enhance our knowledge at the systems level. Domestic cattle (*Bos taurus*) are likely the most-extensively studied ruminant species. Here we present examples of tissue-specific metabolic network reconstructions from human and bovine species. The analysis of tissue-specific pathways and their functional behavior is an integral part of systems biology. This concept as it relates to ruminants has been discussed recently [95] using liver, mammary gland and adipose tissue as an illustration.

A putative cattle genome-wide metabolic pathway assembly was conducted by Seo and Lewin [28] using a bottom-up approach. They essentially applied the comparative analysis approach for the reconstruction process, and observed that between cattle and human metabolic pathways, there was ca. 35% similarity at the enzyme level and 54% similarity at the functional, level with the exception of some differences in individual enzymes and alternative reactions. They also observed that the most-conserved pathways include “energy and nucleotide/nucleoside metabolism,” which are considered to be present in evolutionarily ancient pathways [97].

Genomic approaches may also help to identify previously unrecognized complex biological mechanisms that are unique to ruminants; hence, improving our opportunities for enhancing livestock productivity. Due to the high cost, few nutritional studies with ruminant species have been performed [95]; whereas, more extensive work in this area as it relates to livestock and agriculturally-important species has been conducted using chickens [98]. The high-throughput transcriptomics work conducted to date has greatly expanded our understanding of fundamental molecular mechanisms in ruminants [99, 100]. By analyzing the physiological conditions at critical levels in a ruminant species such as dairy cattle (e.g. lactation, dry period, parturition), in the future we might be able to increase the productive efficiency by optimizing management at the farm level. We and others [101] believe that this can be achieved by obtaining fundamental knowledge of genotypic to phenotypic transitions at the systems level using top-down approaches. Despite the progressive implementation of bioinformatics and systems biology tools in human and microbial species, their applications in livestock species are still in its infancy stages.

DNA microarray and other high-throughput sequencing techniques such as RNA-Seq, are used to measure the expression of the entire transcriptome of an organismin a single or series of experiments. These can detect not only mRNA from highly expressed genes but also from less abundant genes [70, 71, 99]. In fact, RNA-Seq has several advantages over DNA microarrays including the detection of single nucleotide polymorphisms (SNP), alternative splice variants, and RNA editing [102]. These approaches have the ability to unravel genomic information at systems level in contrast to the reductionist paradigm. The resulting data can be used to create networks of genes and/or proteins or to incorporate molecular control points into mechanistic models [101] leading to enhanced knowledge of network biology [103] and overall information at a functional level.

### Tissue-Specific Applications

As indicated above, the genome-scale reconstruction provides a holistic view of an organism; whereas the tissue-specific reconstruction provides a view of metabolic pathways in a tissue-specific manner. Clearly, each tissue has a unique set of metabolic objectives, some of which differ markedly between tissues. Differential expression of genes and proteins in a tissue specific manner plays an important role in determining metabolic fates [104].

Human tissue-specific applications using the systems-biology have been developed by Gille *et al.* [105], Jerby *et al.* [106], and Shlomi *et al.* [104]. For instance, Gille and colleagues [105] reconstructed the human liver using bottom-up constraint-based modeling, which led to development of HepatoNet1. This model has the capability of recreating liver-specific functions, such as cholesterol biosynthesis, bile formation, and ammonia detoxification under optimal conditions. These authors performed FBA on 442 metabolic objective functions to test the liver-specific stoichiometric model as a way to examine hepatic cell behavior. This tissue-specific reconstruction project provided a complete mathematical approach to assess biological functions. The model also allows for evaluating effects of minimal nutritional requirements on pathway behavior. Recently, a tissue-specific metabolic scale-free network using systems biology approaches has also been reconstructed for bovine mammary gland tissue [107].

The biological intricacy of livestock inexorably requires the systematic study of tissue-specific interactions. The above mentioned approaches are equally applicable to the study of tissue-specific transcriptomes. Liver, mammary, and adipose tissue-specific microarray studies have been conducted by our group and others (Table **2**) in the last few years to evaluate the effects of nutrition and physiological state on the transcriptome. This technology allows us to examine the temporal expression of known components of metabolic networks, which is an appropriate means for addressing the issue of transcriptional regulation. This transcriptional regulation is related to tissue-specific metabolism as a response to growth and/or nutritional management in ruminants [108]. To date, more than 46 transcriptome expression profiling research articles using high-throughput genomics techniques on different bovine tissues have been published. (Table **2**) contains information from published articles between 2003 and 2012. The following liver and adipose tissue examples are two particular applications of tissue-specific, top-down reconstructions in cattle (*Bos taurus*).

#### i) Liver

In contrast to tissue-specific bottom-up reconstruction in human hepatocytes, the top-down approach as exemplified by the applications of DNA microarray data has been employed in studies of dairy cattle liver (13 of 46 papers published since 2003, Table **2**). Similar to humans, bovine liver performs a wide range of tissue-specific functions, including cholesterol biosynthesis [109], urea synthesis [110, 111], gluconeogenesis [112], oxidation of non-esterified fatty acids (NEFA), ketogenesis, or esterification of NEFA into triacylglycerol (TAG) [100, 113]. Despite the information generated by these studies, the scope of the bioinformatics analysis based on time-course experiments is quite limited due in part to the reliance on software tools built on the analytical features dealing with overrepresented approach (ORA) [114]. To overcome such limitations, particularly when dealing with time-course or multiple treatment transcriptome data, our group recently has developed and validated a novel DIA analysis [87, 88], which outperforms over the ORA and produces biologically more meaningful interpretation of longitudinal transcriptome data.

We have recently applied DIA analysis to mine the hepatic transcriptome from late pregnancy through early lactation in cows receiving different levels of dietary energy prepartum. For this study, already available DNA microarray data were obtained from NCBI GEO (accession number GSE 3331) [70, 71] and re-analyzed using the Proc MIXED model of SAS. The study was based on two dietary conditions i.e., overfed (OF) versus restricted energy (RE) intake. The tissue biopsies were harvested at days -65, -30, -14, +1, +14, +28, and +49 relative to parturition. A Benjamini-Hochberg FDR correction resulted in a total of 4,111 DEG with a significant diet × time interaction (FDR <0.05). The bioinformatics analysis was carried out using the DIA methodology asdescribed by Bionaz *et al.* [87]. This novel tool uses the information from the KEGG pathway database (http://www.genome.jp/kegg/pathway.html) and can help rank each pathway-based on higher or lower impacted values. In this particular experiment, DIA estimates the overall magnitude of physiological changes (impact) and direction (flux; activation, inhibition, or no change) over time and in response to a dietary treatment.

The Fig. (**4**) contains a set of five highly-impacted pathways obtained from bovine liver data analysis. Among the top affected pathways by plane of nutrition, the five pathways include ubiquinone and other terpenoid-quinone biosynthesis, sulfur metabolism, arachidonic acid metabolism, complement and coagulation cascade and base excision repair. A preliminary interpretation of these results revealed unique responses of bovine liver during transition from pregnancy to lactation. For instance, ubiquinone (coenzyme Q) and other terpenoid-quinone biosynthesis are involved in oxidative phosphorylation as part of the cellular respiratory chain [115], and during the transition into lactation a significant induction was observed in OF cows; while sulfur metabolism was inhibited.

From a biological standpoint, and because its anionic property, the observed adaptation in sulfur metabolism in OF cows might help the liver balance the cation-anion concentration [116]. Metabolism of sulfur also plays a role in the synthesis of sulfur-containing amino acids [117], and indirectly may play a role in lipid metabolism. The activation of arachidonic acid metabolism after parturition in OF cows, i.e. d 1 postpartum, could be related with the synthesis of signaling molecules that may play a role in the overall adaptation of liver to the onset of lactation. Similarly, the inhibition of the complement and coagulation pathway before parturition coupled with its activation at 14 d postpartum in OF cows is an indication that they were more sensitive to mounting an inflammatory response [118]. The gradual activation of the base excision repair pathway between -14 d through 14 d around parturition in OF cows suggested a potentially greater degree of DNA damage because this pathway is central in repairing damaged DNA[119] and the control of cell proliferation [120]. Overall, these results indicate that OF vs. RE prepartum elicited a stronger transcriptional response potentially leading to alterations in immune response, metabolism, and DNA damage. These findings are supported in part by the original studies conducted by Loor *et al.* [71].

#### ii) Adipose Tissue

Relatively fewer transcriptome studies (6 published since 2003, Table **2**) have been conducted on bovine adipose tissue [121-123]. Sumner *et al.* [122] performed transcriptome profiling of subcutaneous adipose tissue during the transition from pregnancy to lactation, and used the ORA approach to mine the DEG. In collaboration with the McNamara group, we used the KEGG-based DIA analysis to evaluate the impact of change in physiological states on biological pathways in bovine adipose tissue. The tissue biopsies were obtained on days -21, -7, +7, and +28 relative to parturition [124]. The ANOVA with an FDR correction resulted in 1,802 DEG with a time effect (FDR < 0.10).

The DIA approach revealed that the onset of lactation resulted in a gradual decrease in the utilization (metabolism) of glucose, lactate, and acetate to produce energy (e.g., most impacted pathways included metabolism of fatty acids, biotin, pyruvate, and TCA cycle) [124]. Furthermore fatty acid desaturation, elongation, and PPAR signaling were markedly inhibited during lactation. Among the significantly-affected, the complement and coagulation cascade pathway of the immune system also was induced. While implementing the DIA using the DAVID bioinformatics resources, it was observed that fatty acid biosynthesis, linoleic acid metabolism, biotin metabolism, and glycerolipid metabolism were markedly inhibited postpartum than prepartum; whereas, complement and coagulation cascades and riboflavin metabolism were among the only pathways with sustained induction postpartum relative to prepartum.

Overall, the preliminary evaluation of the combined results from both bioinformatics approaches indicated that the adipogenic capacity of adipose tissue is quite robust during late pregnancy while the innate immune response of the tissue is more predominant during early lactation. The latter may be a response of the tissue due to stressors such as cytokines/hepatokines, NEFA, and/or pathogens. Alternatively, it may represent a mechanism associated with tissue remodeling [124]. The liver and adipose-specific applications provide evidence that systems biology approaches inevitably lead to a better understanding of the functional changes in an organism due to internal or external factors.

## CONCLUDING REMARKS AND FUTURE CHALLENGES

The primary objective of this review was to provide a concise overview of the evolution of systems biology approaches and its potential applications in ruminants using transcriptomic data. To enhance our understanding of the complex biological behavior in ruminants, there is a need for integration of genome-enabled and computational techniques. Work during the previous 15 years on model organisms has clearly demonstrated the applicability of high-throughput technologies coupled with genome-scale models to elucidate systematic interactions [125].

Bottom-up systems biology deals with the known stoichiometry of chemical reactions in biological systems by means of labor-intensive literature surveys and computational resources *in silico*. There is a substantial body of work on biochemical pathways and their regulation in the ruminant animal [25]. That information will prove useful when applying the bottom-up approach within the systems framework. However, the bottom-up approach leaves some gaps in genome-scale models because of our incomplete knowledge in non-model organisms such as cattle. These gaps could be filled by using conserved evolutionary relationships among species. Top-down systems biology examines molecular interactions in complex biological systems through genome-wide ‘omics’ studies. As part of this approach we can uncover relationships among genes and proteins, but more importantly, among biological networks.

Both approaches are complimentary in the search for interrelationships between genotypes and phenotypes. With the availability of tissue-specific genome-scale models constructed from ‘omics’ data and already published research articles, our understanding of the impact of genomic background on an observed phenotype will be enhanced. Ultimately, these models will help to explain diverse molecular interactions among various networks, from the cellular level up to the organism level in an integrative manner [126]. It is also worth mentioning that both reductionist and integrative approaches can help describe the functional behavior of a cell [103].

Even though much progress has taken place in ‘omics’, bioinformatics, and systems biology, its specific applications in ruminants are still minor relative to model organisms [95]. To accelerate progress in ruminant systems biology, there is a need for automation to help handle the growing number of datasets originating from genome-enabled tools. The application of modern computational resources in ruminant biology can improve our understanding about molecular interactions *in silico*. Over the long term, the end result of this work could help to improve productive performance, and ultimately lead to more efficient ways of managing dairy cattle for production of milk and meat to meet the demands for highly nutritious food for humans worldwide.

## Figures and Tables

**Fig. (1):**
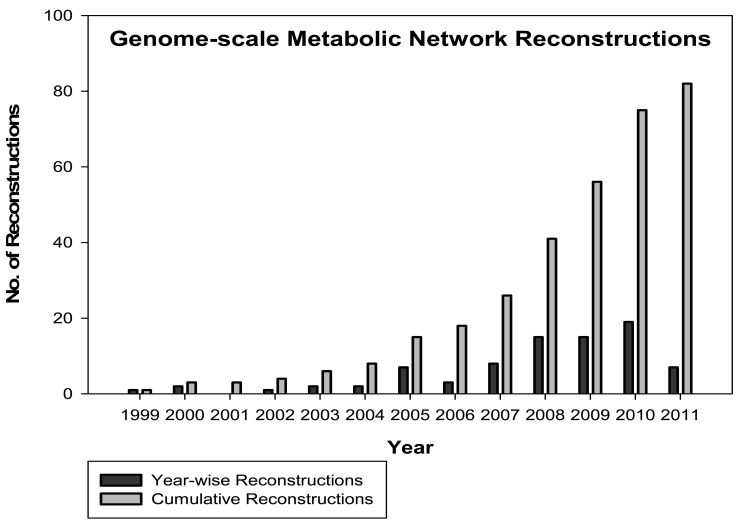
Genome-scale metabolic network reconstructions statistics from 1999 to 2011. Year-wise (red) and cumulative (green) studies with respect to total number of reconstructions. The data include a wide range of species from bacteria to eukaryotes.

**Fig. (2):**
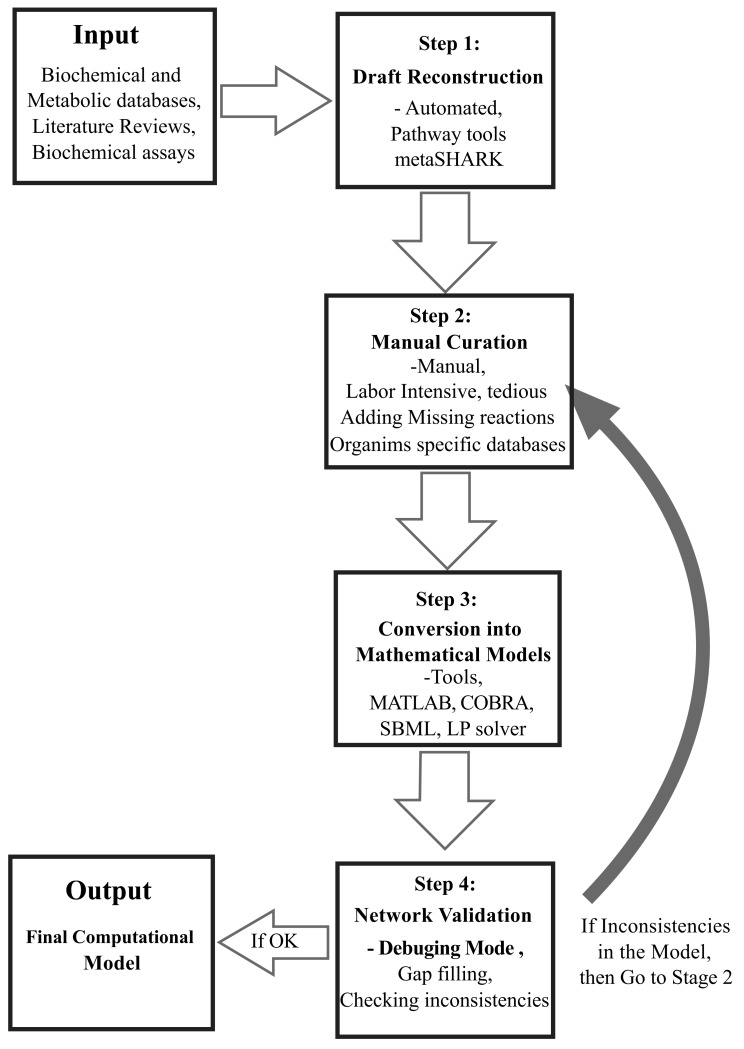
bottom-up systems biology approach. The four conventional steps of modern systems biology are summarized in the figure. Information obtained from biochemical and metabolic databases is given as an input to start building the genome-scale computational models. Step 1 deals with the automated draft reconstruction bioinformatics tools such as pathway tools and metaSHARK. This first stage still leaves some gaps, missing reactions, and deadend metabolites (i.e., metabolites having unknown reactants or product information). Once the automated draft is created, it needs manual curation, which is completed during step 2. This step involves consulting through organism-specific databases, adding missing reactions, and dealing with dead-end metabolites. Step 3 involves the conversion of the refined draft into mathematical models using stoichiometric calculations. This step involves the application of Matlab-embedded tools (e.g., COBRA, SBML) and linear/ quadratic programing solvers to create mathematical models and allows visualization of results on the Matlab interface. Step 4 involves the simulation and evaluation of the reconstructed genome- scale mathematical models under optimal conditions. If there are some inconsistencies in the model, then it is re-evaluated from Step 2. If the model is working correctly in the final stage, then it is considered for further computational applications.

**Fig. (3):**
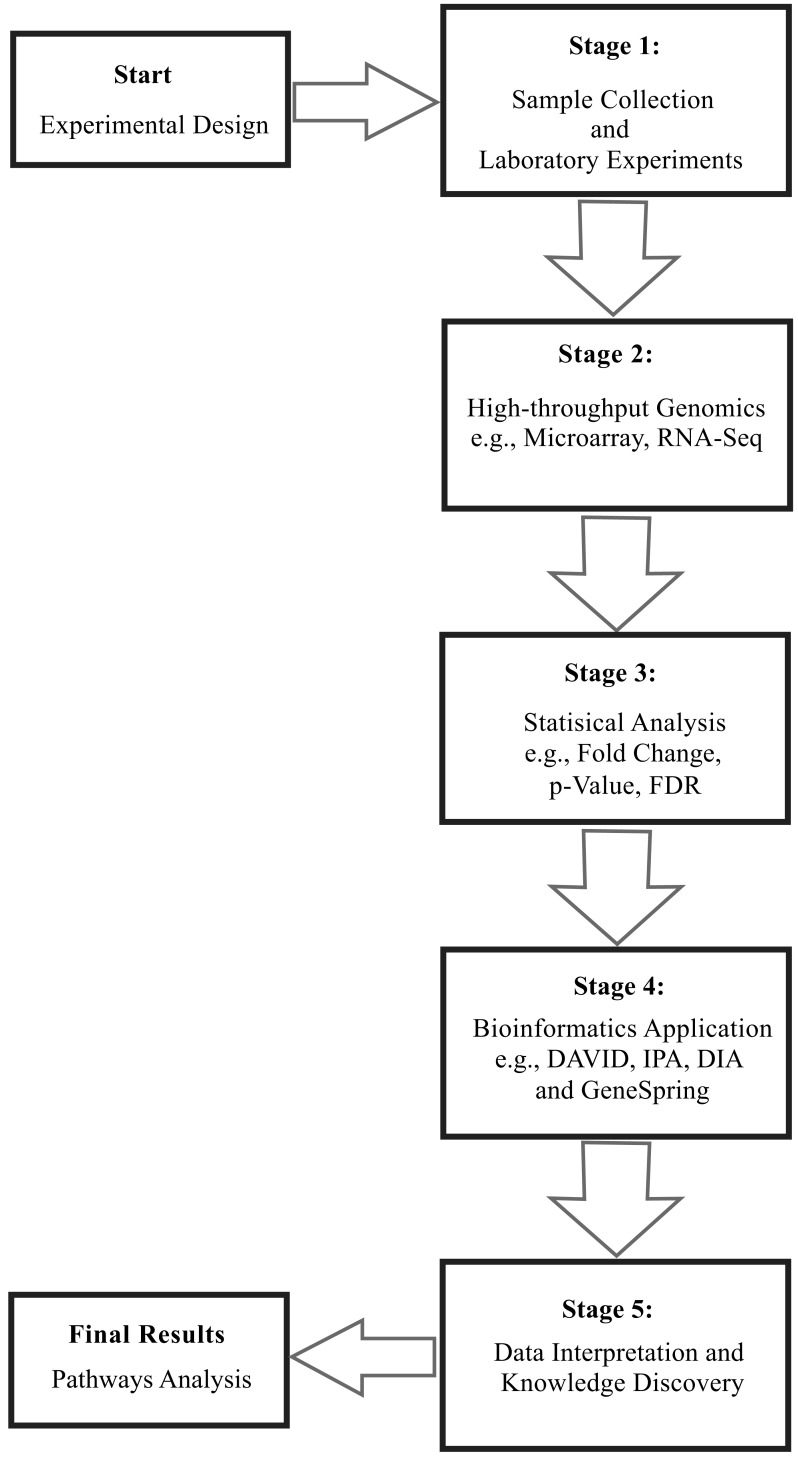
Top-down systems biology approach. This approach is categorized into five main stages. After designing an experiment, the first stage involves biological sample collection (e.g. tissue biopsy) of control and treated animals. This is followed by laboratory experiments including RNA extraction, purification, and expression profiling. Stage 2 involves high-throughput genomics using microarray platforms (e.g. Affymetrix) and RNA-Seq. Stage 3 involves data normalization to remove noise and obtain high-quality expression profiling data for statistical analysis utilizing suitable tools (e.g. SAS) and incorporating the key aspects of the experimental design (e.g. time, treatment, and any potential interactions). After the statistical tests, differential expression is determined based on a certain p-value criterion. In the stage 4 the significant data are analyzed through bioinformatics techniques. The last stage involves data interpretation and knowledge discovery leading towards the development of new scientific hypothesis.

**Fig. (4):**
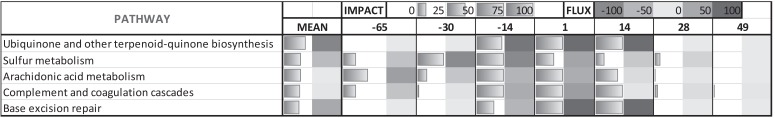
Top 5 impacted pathways sorted by overall impact in response to overfeeding (OF) versus restricting dietary energy (RE) during the prepartum period in dairy cattle. The data correspond to days -65, -30, -14, +1, +14, +28 and +49 relative to parturition. The impact values are shown in light-blue colored horizontal bars (from 0 to 100 based on the biological perturbation in a pathway), while flux values are depicted in red (activated/induced) to green (inhibited/reduced) shades of color (-100 to 100). The impact corresponds to the overall perturbation in a pathway while flux corresponds to the direction of the impact. The “mean” column represents the overall change of impact and flux from day -65 to day +49.

**Table 1.** List of Bioinformatics Software Commonly Used for Data Mining and Analysis in Ruminant Research. The Reference Column Provides Selected Examples of Published Studies that have used these tools

Sr. #	Name	Link	Reference
1.	DAVID	http://david.abcc.ncifcrf.gov/	[92]
2.	GeneSpring GX	http://www.genomics.agilent.com/	[70, 71]
3.	IPA	http://ingenuity.com/	[84, 127]
4.	Genesis	http://genome.tugraz.at/genesisclient/genesisclient_description.shtml	[86]
5.	KEGG	http://www.genome.jp/kegg/	[92]
6.	DIA	Dynamic Impact Approach	[88]
7.	MetaCore	http://www.genego.com/metacore.php	[128]
8.	GOseq	http://www.bioconductor.org/packages/2.9/bioc/html/goseq.html	[129]

**Table 2.** Published Bovine Studies Between 2003-2012 Using High-Throughput Genomics Technologies

Title	Year	Tissue(s)	Technology Used	Reference
“Bovine mammary gene expression profiling using a cDNA microarray enhanced for mammary-specific transcripts	2003	Mammary	DNA Microarray	[130]
“Generation of a bovine oocyte cDNA library and microarray: resources for identification of genes important for follicular development and early embryogenesis	2004	Fetal ovary	DNA Microarray	[131]
“Transcriptional profiling of skeletal muscle tissue from two breeds of cattle	2004	Skeletal muscle	DNA Microarray	[132]
“Pregnancy-associated changes in genome-wide gene expression profiles in the liver of cow throughout pregnancy	2004	Liver	DNA Microarray	[133]
“Temporal gene expression profiling of liver from periparturient dairy cows reveals complex adaptive mechanisms in hepatic function	2005	Liver	DNA Microarray	[70]
“Plane of nutrition prepartum alters hepatic gene expression and function in dairy cows as assessed by longitudinal transcript and metabolic profiling	2006	Liver	DNA Microarray	[71]
“Developmental aberrations of liver gene expression in bovine fetuses derived from somatic cell nuclear transplantation	2006	Fetal liver	DNA Microarray	[134]
“Identification of estrogen-responsive genes in the parenchyma and fat pad of the bovine mammary gland by microarray analysis	2006	Mammary	DNA Microarray	[135]
“A gene coexpression network for bovine skeletal muscle inferred from microarray data	2006	Skeletal muscle and adipose	DNA Microarray	[136]
“Nutrition-induced ketosis alters metabolic and signaling gene networks in liver of periparturient dairy cows	2007	Liver	DNA Microarray	[84]
“Target genes of myostatin loss-of-function in muscles of late bovine fetuses	2007	Muscle	DNA Microarray	[137]
“Image analysis and data normalization procedures are crucial for microarray analyses	2008	Muscle and adipose	DNA Microarray	[138]
“Gene expression patterns during intramuscular fat development in cattle	2008	Muscle and lean mass (LM) tissue	DNA Microarray	[139]
“Comparative proteomics and transcriptomics analyses of livers from two different Bos taurus breeds: "Chianina and Holstein Friesian"	2009	Liver	DNA Microarray	[140]
“Pleiotropic effects of negative energy balance in the postpartum dairy cow on splenic gene expression: repercussions for innate and adaptive immunity	2009	Spleen	Affymetrix GeneChip Bovine Genome Array	[141]
“Feasibility of a liver transcriptomics approach to assess bovine treatment with the prohormone dehydroepiandrosterone (DHEA)	2010	Liver	DNA Microarray	[142]
“Negative energy balance and hepatic gene expression patterns in high-yielding dairy cows during the early postpartum period: a global approach	2010	Liver	Affymetrix GeneChip Bovine Genome Array	[143]
“Dietary supplementation of selenium in inorganic and organic forms differentially and commonly alters blood and liver selenium concentrations and liver gene expression profiles of growing beef heifers	2010	Liver	DNA Microarray	[144]
“Effect of diet supplementation on the expression of bovine genes associated with fatty acid synthesis and metabolism	2010	Adipose	Affymetrix GeneChip Bovine Genome Array	[145]
“Omega-6 fat supplementation alters lipogenic gene expression in bovine subcutaneous adipose tissue	2010	Adipose	DNA Microarray	[146]
“Altered gene expression in human adipose stem cells cultured with fetal bovine serum compared to human supplements	2010	Adipose	DNA Microarray	[147]
“Microarray analysis of gene expression profiles in the bovine mammary gland during lactation	2010	Mammary	Affymetrix GeneChip Bovine Genome Array	[148]
“Enhanced mitochondrial complex gene function and reduced liver size may mediate improved feed efficiency of beef cattle during compensatory growth	2010	Liver	DNA Microarray	[149]
“Transcriptomic profiling of bovine IVF embryos revealed candidate genes and pathways involved in early embryonic development	2010	IVF-derived blastocysts and embryos	DNA microarray	[150]
“Comparison of transcriptomic landscapes of bovine embryos using RNA-Seq	2010	Embryos	RNA-Seq	[151]
“SNP discovery in the bovine milk transcriptome using RNA-Seq technology	2010	Milk somatic cells	RNA-Seq	[152]
“Characterization of the abomasal transcriptome for mechanisms of resistance to gastrointestinal nematodes in cattle	2011	Fundic abomasum	RNA-Seq	[153]
“Indistinguishable transcriptional profiles between *in vitro*- and *in vivo*-produced bovine fetuses	2011	Liver and placenta	DNA Microarray	[154]
“Global gene expression profiling reveals genes expressed differentially in cattle with high and low residual feed intake	2011	Liver	DNA Microarray	[155]
“Gene expression differences in oocytes derived from adult and prepubertal japanese blackcattleduring *in vitro* maturation	2011	Oocytes	Microarraygene chips	[156]
“Microarray analysis of differentially expressed microRNAs in non-regressed and regressed bovine corpus luteum tissue; microRNA-378 may suppress luteal cell apoptosis by targeting the interferon gamma receptor 1 gene	2011	Corpus luteum	miRNA microarray	[157]
“Transcriptome profiling of bovine milk oligosaccharide metabolism genes using RNA-sequencing	2011	Milk somatic cells	RNA-Seq	[158]
“Gene expression in the arcuate nucleus of heifers is affected by controlled intake of high- and low-concentrate diets	2012	Brain	DNA Microarray	[159]
“Endometrial gene expression during early pregnancy differs between fertile and subfertile dairy cow strains	2012	Endometrial tissue	DNA Microarray	[160]
“Gene expression profiling of bovine peripartal placentomes: detection of molecular pathways potentially involved in the release of foetal membranes	2012	Placentomes	Affymetrix GeneChipBovineGenome Array	[161]
“Muscle transcriptomic analyses in angus cattle with divergent tenderness	2012	Muscle	Microarray	[162]
“Transcriptome analysis of subcutaneous adipose tissues in beef cattle using 3' digital gene expression-tag profiling	2012	Subcutaneous adipose tissue (backfat)	Digital gene expression-tag profiling	[163]
“Level of nutrient intake affects mammary gland gene expression profiles in preweaned Holstein heifers	2012	Mammary	DNA microarray	[164]
“Reconstruction of metabolic network in the bovine mammary gland tissue	2012	Mammary	DNA Microarray	[107]
“Cytoskeleton remodeling and alterations in smooth muscle contractility in the bovine jejunum during nematode infection	2012	Jejunum	RNA-Seq	[165]
“Characterization of the longissimus lumborum transcriptome response to adding propionate to the diet of growing Angus beef steers	2012	Longissimus lumborum muscle	RNA-Seq	[102]
“Conceptus-endometrium crosstalk during maternal recognition of pregnancy in cattle	2012	Endometrium tissues	RNA-Seq	[166]
“RNA-Seq analysis uncovers transcriptomic variations between morphologically similar *in vivo*- and *in vitro*-derived bovine blastocysts	2012	Blastocysts	RNA-Seq	[167]
“Effect of the metabolic environment at key stages of follicle development in cattle: focus on steroid biosynthesis	2012	Ovarian follicle	RNA-Seq	[127]
“Transcriptional profiling of bovine milk using RNA sequencing	2012	Milk somatic cells	RNA-Seq	[128]
“RNA-seq analysis of differential gene expression in liver from lactating dairy cows divergent in negative energy balance	2012	Liver	RNA-Seq	[129]
“Characterization and comparison of the leukocyte transcriptomes of three cattle breeds	2012	Leukocytes	mRNA-Seq	[168]
“Perturbation dynamics of the rumen microbiota in response to exogenous butyrate	2012	Rumen epithelium	Pyrosequencing	[169]
